# Freezing resistance evaluation of rose stems during frost dehardening using electrical impedance tomography

**DOI:** 10.1186/s12870-021-02976-w

**Published:** 2021-04-26

**Authors:** Ji Qian, Juan Zhou, Ruijuan Gong, Yang Liu, Gang Zhang

**Affiliations:** 1 College of Horticulture, Hebei Agriculture University, Baoding, 071000 Hebei China; 2 College of Electrical and Mechanical Engineering, Hebei Agricultural University, Baoding, 071000 Hebei China; 3 College of Art, Hebei University of Economics and Business, Shijiazhuang, 050051 Hebei China; 4 Department of Software Engineering, Hebei Software Institute, Baoding, 071000 Hebei China

**Keywords:** Electrical impedance tomography, Electrolyte leakage, Image processing, Image reconstruction, Plant freezing resistance

## Abstract

**Background:**

Electrical impedance tomography (EIT) has rarely been applied in plant science, particularly to study plant resistance to abiotic and biotic stresses. In this study, we evaluated the freezing resistance of floribunda roses (*Rosa* Floribunda) during frost dehardening using the EIT technique to identify a new method for rapid and non-destructive measurement of plant freezing resistance.

**Results:**

The current was the excitation source, the boundary voltage value was measured, and then the boundary voltage reconstructed value was formed. Using an imaging algorithm, the two-dimensional (2D) distribution of impedance or impedance variation was reconstructed. The EIT reconstructed values decreased obviously with the decline in freezing temperatures. The EIT reconstructed values of stems had the best fit to the logistic equation, and subsequently, the semi-lethal temperatures were calculated. The freezing resistance results evaluated using EIT reconstructed values were linearly correlated with the results of the traditional electrolyte leakage (EL) method (*r* = 0.93, *P* < 0.01).

**Conclusions:**

In conclusion, after freezing tests, the reconstructed values of EIT images could be used to quantitatively evaluate the freezing resistance of floribunda rose stems. The present study provides a reference for the further application of the EIT technique for non-destructive and rapid detection of plant freezing resistance.

## Background

The ability to survive sub-freezing temperatures is the single most important factor limiting the distribution of many tree species and affecting woody plant growth, development and many genetic, physiological and biochemical responses [1, 2]. To obtain the correct information of changes in freezing resistance in different stages of the annual growth cycle of woody plants, precise and reliable methods for the estimation of freezing resistance are extremely important for a tree species or variety and for cultivation potential evaluations. These methods are also needed in breeding and selection work and in studies of the mechanisms of frost injury, frost hardening and dehardening. Freezing resistance is usually measured by exposing plant tissues or organs to controlled freezing temperatures and then quantifying tissue damage by one or more methods. Research on the identification and testing of freezing resistance has so far achieved considerable results and experience, but there is still room for progress [2]. Steponkus and Lanphea [3] proposed the definition of an ideal method of testing for freezing resistance. However, application of the measures for studying, evaluating, and identifying freezing resistance is constrained by many factors, such as tree species, organs, tissue types, and physiological conditions of trees, as well as research objectives and instruments. Since 19th-century studies of freezing resistance in plants, the methods to measure freezing resistance have involved traditional direct field observations such as visually scoring damage and using the electrolyte leakage method; In recent years, Aslamarz A. A. and other researchers have used Triphenyl Tetrazolium Chloride Assay (TTC), Proline Analysis and Computer Assisted Thermal Analysis (TA) to study the cold tolerance of plants [4–7]. However, these methods have time-consuming and laborious drawbacks, and several days are required to complete the determination of freezing resistance. Thus far, no ideal method has been widely accepted as a reliable and efficient means of testing plant freezing resistance. Improving and innovating the identification and test method of freezing resistance is essential and will provide a significant direction for studies on plant freezing resistant physiology. To achieve such an ideal method, studies based on the existing method and the development of a new method are great challenges for freezing resistance researchers.

In 1978, Henderson and Webster [8] applied the Electrical Impedance Tomography (EIT) technique to the study of pulmonary oedema and generated the first impedance image. In recent years, the EIT technique has developed rapidly, some researchers have used EIT technology to study the medical field, soil properties, wood decay and phenotypic information of plant roots [9–28]. Corona-Lopez [14] and Li, X et al. [15] conducted root in situ detection on the soil-root system to obtain the sectional images of the roots of trees and seeds of *Brassica napus* L. (Variety Temple), and to determine the morphological information of the roots. But the EIT technique has rarely been applied in plant resistance to abiotic and biotic stresses. According to Bera [21], under alternating electrical excitation, biological tissues produce complex electrical impedance that depends on tissue composition, structures, health status, and applied signal frequency, and hence the bioelectrical impedance methods can be utilized for non-invasive tissue characterization. Over the past few decades, a number of impedance-based non-invasive tissue characterization techniques such as EIT have been proposed, and numerous studies have been conducted using these methods for non-invasive tissue characterization and disease diagnosis. Thus, EIT might be used to realize real-time monitoring of plant resistance without damaging the plant.

**Fig. 1:**
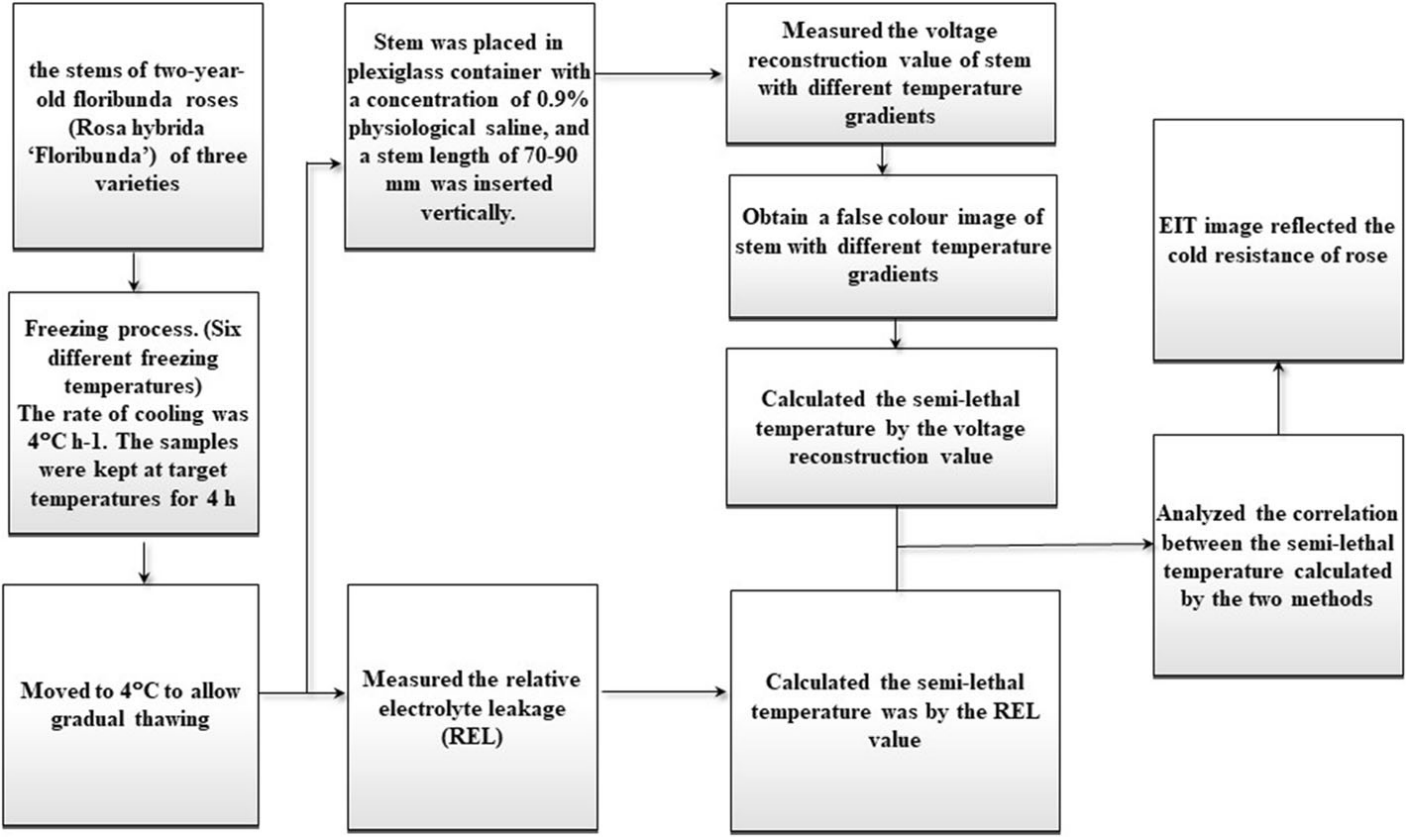
Freezing resistance evaluation protocol of rose stems during frost dehardening using electrical impedance tomography

In the present study, the EIT technique was used to evaluate the freezing resistance of three floribunda rose (*Rosa* Floribunda) varieties under a controlled freezing test during frost dehardening. The semi-lethal temperature (LT_50_, temperature at which 50% of the plants are killed) was calculated and compared with that obtained by the traditional electrolyte leakage (EL) method. The freezing resistance among varieties was analysed using EIT with the aim of identifying a new technique for rapid and non-destructive detection of freezing resistance in plants and for application to plant resistance, breeding and other fields. We hypothesized that the EIT images of plants are altered under low temperature stress. With this technique, we used the current as the excitation, measured the boundary voltage value, and then form the boundary voltage reconstructed value. The distribution of electrical impedance inside the plant was calculated using an imaging algorithm. Then, the two-dimensional (2D) distribution of impedance or impedance variation was reconstructed. Under low temperature stress, the expected resistivity will decrease; the freezing resistance of plants was characterized directly and quantitatively by image reconstruction (Fig. 1).

## Results

### Evaluation of EIT reconstruction quality

**Fig. 2:**
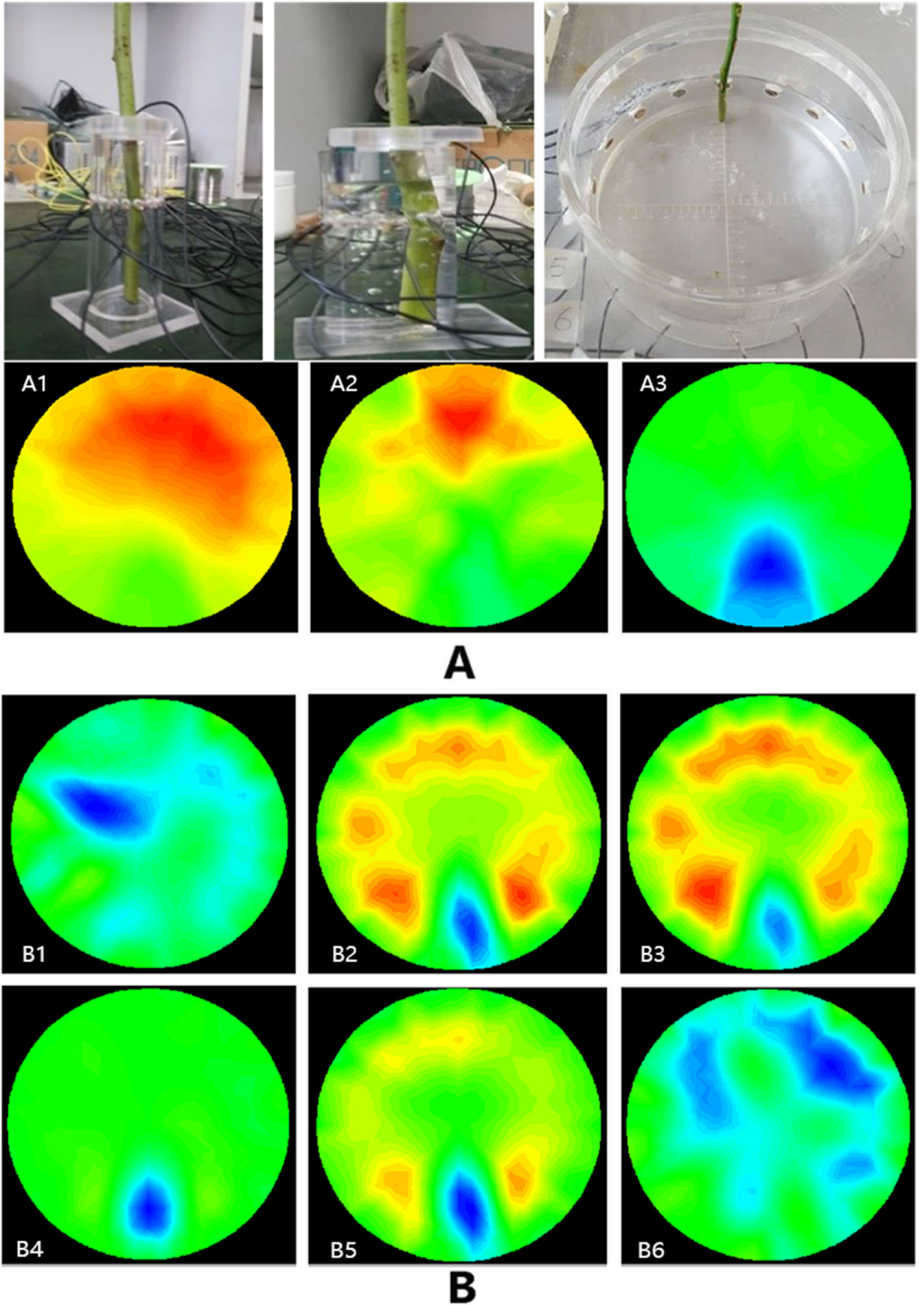
**a** Reconstructed images with different test devices and **b** reconstructed images with different excitation currents The radius of the test device was 2 cm (A1), 4 cm (A2) or 8 cm (A3). The excitation current was 100 μA (B1), 150 μA (B2), 200 μA (B3), 250 μA (B4), 300 μA (B5) or 500 μA (B6). The diameter of the rose stem was 5.68 mm, and the excitation current frequency was 1 kHz. The excitation current was 250 μA for **a**, and the radius of the test device was 8 cm for **b**. The blue area on the lower part of each image represented the stem of the rose, the green area represented the background salt solution, and the other colours indicated that serious noise occurred.

Based on the brain model of the Laboratory of Biomedical Engineering, the Fourth Military Medical University and combined with the present experimental plant material, a test device with a plexiglass container with a radius of 2, 4 or 8 cm was customized at Tsinghua University, and 16 electrodes were placed equidistantly on the wall of the device (Fig. 2a).

**Fig. 3:**
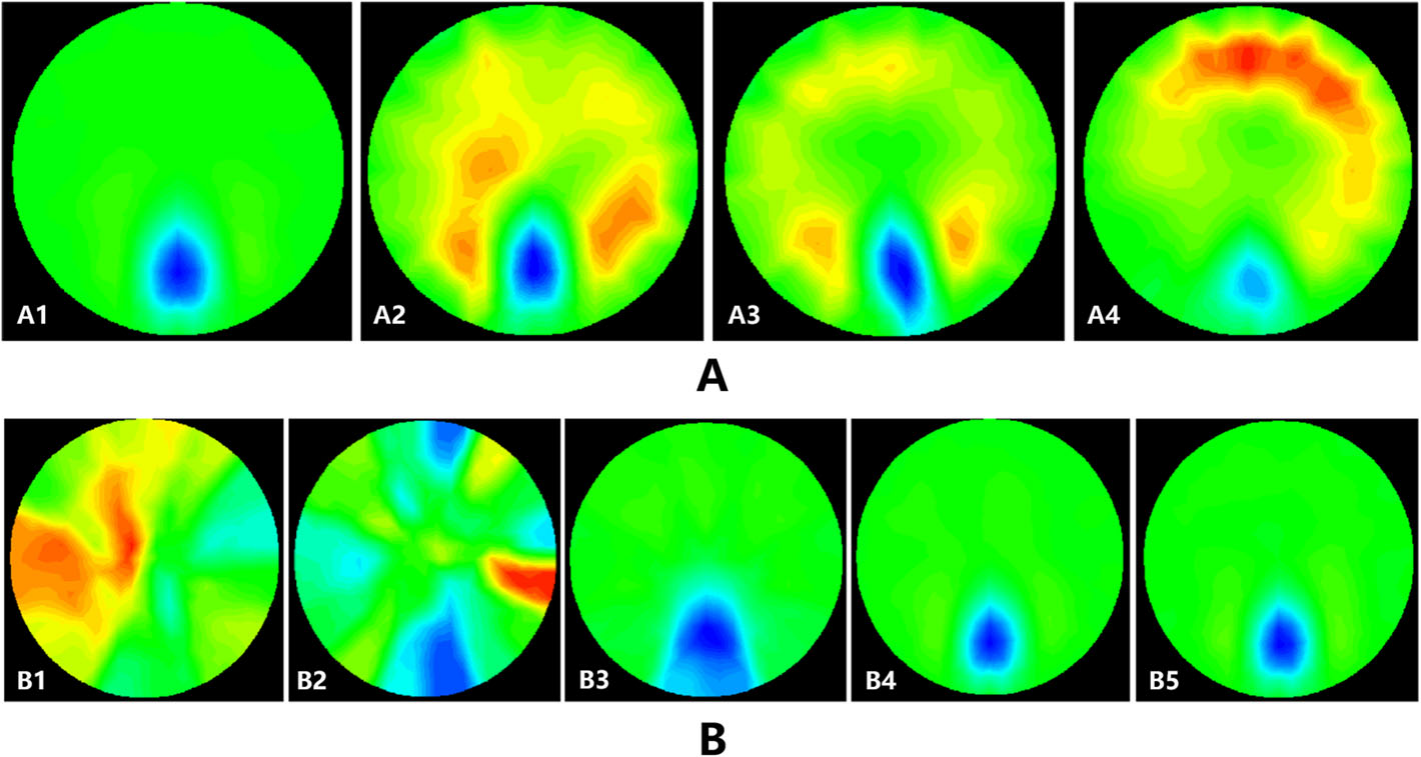
**a Reconstructed images with different excitation current frequencies.** The frequency was 1 KHz (A1), 50 KHz (A2), 100 KHz (A3) or 150 KHz (A4). The rose stem diameter was 5.68 mm, and the excitation current was 250 μA. **b Reconstructed images with different diameters of rose stems.** The diameter of rose stems was 2.45 mm (B1), 3.23 mm (B2), 4.44 mm (B3), 5.63 mm (B4) or 6.29 mm (B5). The excitation current was 250 μA, the excitation frequency was 1 kHz. The radius of the test device was 8 cm. The blue area on the lower part of each image represented the stem of the rose, the green area represented the background salt solution, and the other colours indicated that serious noise occurred

To determine the test parameters used for the freezing resistance measurement, 5 different rose stem diameters of approximately 2, 3, 4, 5, and 6 mm; 6 different excitation current intensities of 100, 150, 200, 250, 300, and 500 μA (Fig. 2b); and 4 different current frequencies of 1, 50, 100, and 150 kHz were applied (Fig. 3a).

In the physical model, the experimental parameters of the best reconstructed image were obtained when the size and frequency of the excitation current were 250 μA and 1 kHz, respectively (Figs. 2, 3b, a), the inner diameter of the test device radius was 8 cm and the diameter of the rose stems was greater than 4 mm (Figs. 3b).

Serious noise occurred in the reconstructed image, and the measured stem of the rose could not be clearly distinguished when the device had a radius of 2 or 4 cm (Fig. 2 A1, A2), whereas the measured stem could be distinguished in the image when the device had a radius of 8 cm (Fig. 2 A3). When the electrode spacing was greater than 1 cm, the stem size, shape and position could be obtained from the reconstructed image shown in Fig. 2 A3.

When the diameter of the rose stem was 5.68 mm, i.e., larger than 4 mm, and the excitation current frequency was 1 kHz, the reconstructed image changed with the magnitude of the excitation current (Fig. 2b). The reconstructed image was optimum when the excitation current was 250 μA (Fig. 2 B4).

When the rose stem diameter was 5.68 mm and the excitation current was 250 μA, the reconstructed image changed with the excitation current frequency (Fig. 3a). The reconstructed image was optimum when the excitation current frequency was 1 kHz (Fig. 3 A1).

The different stem diameters of roses were 2.45 mm, 3.23 mm, 4.44 mm, 5.63 mm and 6.29 mm. Each experiment was conducted in the same position with an excitation current of 250 μA and an excitation frequency of 1 kHz. When the stem diameter was 2.45 mm (Fig. 3 B1) and 3.23 mm (Fig. 3 B2), the reconstructed images could not be generated because the stem diameter of the rose was too small. When the stem diameter of the rose was larger than 4 mm (Fig. 3 B3, B4, B5), a clearer EIT image of the stem was detected, and the stem size, shape and position were obtained.

### EIT image and reconstructed values of floribunda rose stems during frost dehardening

**Fig. 4:**
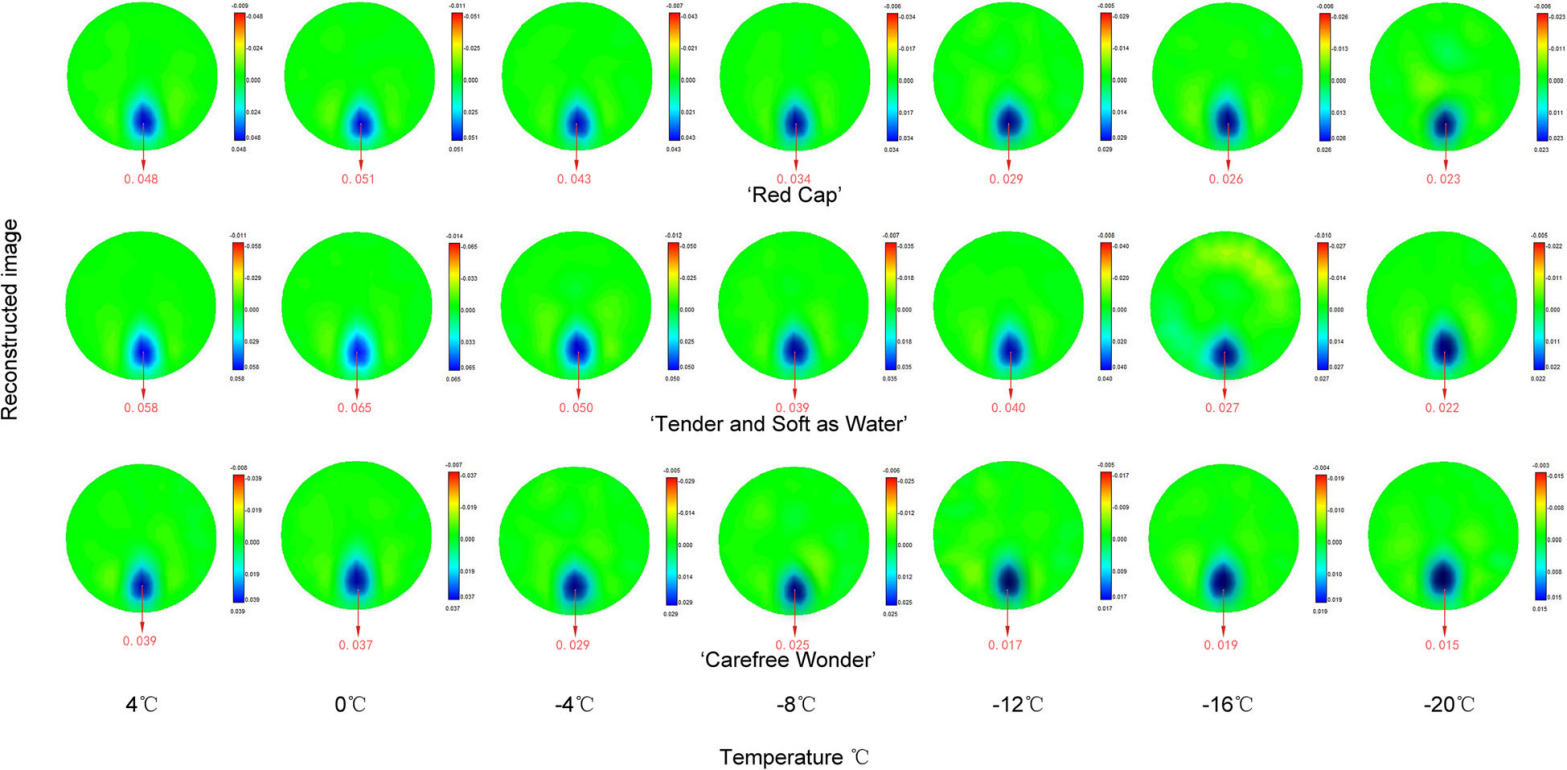
Electrical impedance tomography (EIT) images of stems of three floribunda rose varieties after a controlled freezing test on 22 February. The colour bars on the right side of each image represent the reconstructed values (relative values with a range between 0 and 0.1). The red color numbers under each image represent the largest value of the reconstructed value on each freezing temperature. They are not in the original images and are inserted numbers.

**Fig. 5:**
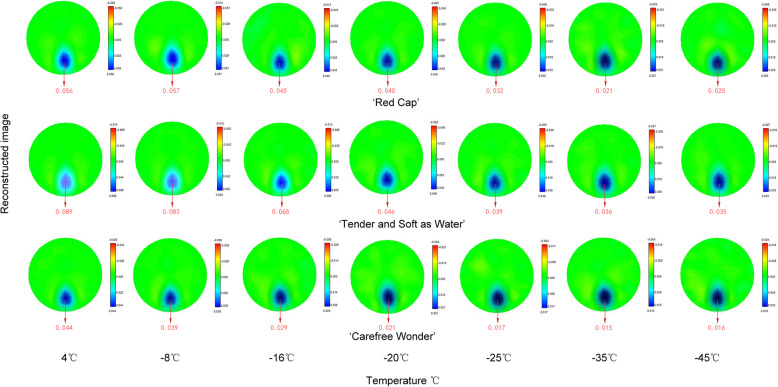
Electrical impedance tomography (EIT) images of stems of three floribunda rose varieties after a controlled freezing test on 16 May. The colour bars on the right side of each image represent the reconstructed values (relative values with a range between 0 and 0.1). The red color numbers under each image represent the largest value of the reconstructed value on each freezing temperature. They are not in the original images and are inserted numbers.

**Fig. 6:**
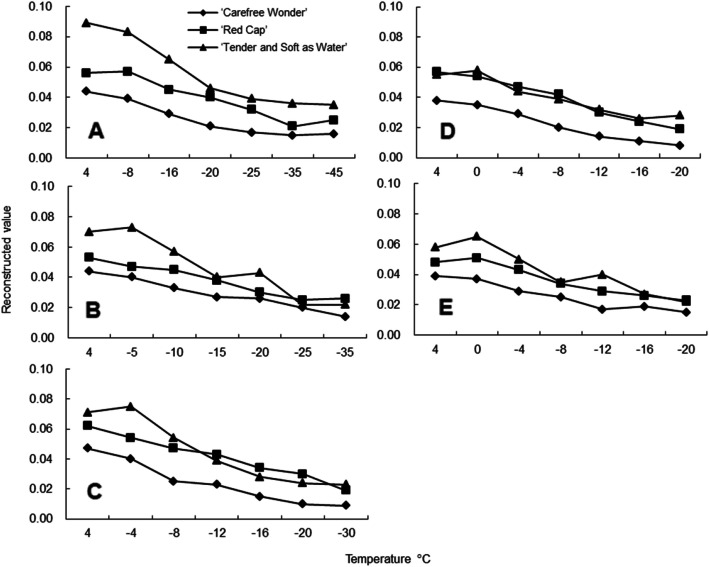
Changes in the electrical impedance tomography (EIT) reconstructed value of stems of three floribunda rose varieties after a controlled freezing test on 22 February **a**, 14 March **b**, 4 April **c**, 25 April **d**, and 16 May **e**. The reconstructed values are relative values with a range between 0 and 0.1.

One frame of EIT images was captured at each temperature for each of the three floribundas rose varieties at each sampling time (Figs. 4, 5). From these images, during frost dehardening, the EIT imaging accurately represented the position and the size of the rose stems. The position of stems presented blue for which the reconstructed value was greater than 0, indicating that the resistivity of the region was increased, and the pure blue in each EIT image corresponded to the maximum value of the reconstruction result. Clear EIT images were obtained throughout the frost dehardening period. With the decrease in freezing temperature, the EIT reconstructed values of the stems showed a decreasing trend, and the values of ‘Carefree Wonder’ were between 0.044 and 0.015, which were smaller than those of the other two cultivars at the 5 sampling times (Fig. 6).

### Changes in freezing resistance

#### Freezing resistance evaluated by means of the EIT reconstructed values and EL method values

During frost dehardening, the freezing resistance of the stems of the rose varieties measured by the EL method showed an overall decreasing trend (Table 1). In the early stage of frost dehardening (February), the order of freezing resistance was ‘Tender and Soft as Water’ > ‘Red Cap’ > ‘Carefree Wonder’. During the period from 14 March to 25 April, the freezing resistance of each variety decreased rapidly, and the order of freezing resistance was ‘Red Cap’ > ‘Tender and Soft as Water’ > ‘Carefree Wonder’. The result of freezing resistance on 16 May was higher than that of the other periods; however, the freezing resistance did not differ significantly among the three varieties (Table 1).

**Table 1:** Comparison of freezing resistance (FR, LT_50_) in stems of three floribunda rose varieties measured by means of relative electrolyte leakage (REL) and electrical impedance tomography reconstructed values (EIT-RV) (The calculations of FR (LT_50_) by different methods see “Freezing resistance calculation by means of EIT reconstructed values and EL method values” in detail)

Varieties	Date (Month- Day)	FR (LT_50_) (°C)					
		REL (95% Confidence interval) EIT-RV (95% Confidence interval)					
‘Red Cap’	2–22	−18.8	−20.36	− 17.21	− 20.1	− 24.74	− 15.42
	3–14	−18.4	− 20.01	− 16.85	− 14.6	−19.14	− 10.15
	4–4	−12.4	− 12.97	− 11.82	− 11.8	− 16.83	−6.69
	4–25	−8.9	−10.06	− 7.82	− 7.3	−32.24	17.59
	5–16	−9.9	−11.08	−8.75	− 5.0^*^	− 8.01	−2.01
‘Tender and	2–22	−21.4	−22.28	− 20.51	− 16.0^*^	− 17.14	− 14.79
Soft as	3–14	−17.6	− 18.79	− 16.47	− 13.6	−24.14	−3.10
Water’	4–4	−11.9	−12.20	− 11.53	−9.9	− 12.61	− 7.29
	4–25	−8.5	−27.28	10.19	− 6.1	− 12.09	− 0.05
	5–16	− 9.4	− 10.51	− 8.25	−8.2	− 15.95	− 0.46
‘Carefree	2–22	−17.3	− 18.17	−16.45	− 15.8	− 16.78	− 14.81
Wonder’	3–14	− 17.1	− 17.98	− 16.22	− 12.7	− 18.85	− 6.51
	4–4	−10.5	−11.29	− 9.71	− 6.9	− 15.12	1.30
	4–25	− 8.6	− 9.53	−7.67	−6.7	−9.05	− 4.30
	5–16	−9.5	− 10.14	−8.81	− 5.1	−10.74	0.44

The row data with an asterisk (*) show a significant difference (*P* < 0.05) in freezing resistance with result of the REL method. The differences (*P* < 0.05) between each row data in freezing resistance of EIT method with results of the REL method for the semi-lethal temperature (LT_50_, Temperature at which 50% of the plants or organs are killed) for each time instant were considered significant when the values of the Wald 95% confidence intervals did not overlap

The LT_50_ was well fitted by EIT reconstructed values and the logistic equation (*R*^2^ > 0.90), and only two significant differences were detected between the LT_50_ obtained by the EIT reconstructed values and the EL method (Table 1). On 25 April, the freezing resistance of ‘Tender and Soft as Water’ was lowest at − 6.0 °C, whereas ‘Red Cap’ and ‘Carefree Wonder’ had the lowest freezing resistance on 16 May, i.e., − 7.0 °C and − 5.0 °C, respectively. Overall, the order of freezing resistance was ‘Red Cap’ > ‘Tender and Soft as Water’ > ‘Carefree Wonder’ (Table 1).

During frost dehardening, the EIT reconstructed values could be used to obtain the freezing resistance of rose stems after controlled freezing tests. Although the LT_50_ values of stems calculated by EIT reconstructed values were higher than those obtained using the EL method, no significant differences occurred between the results. (Table 1).

#### Comparison and validation of freezing resistance in stems evaluated by EIT reconstructed values with EL method

**Fig. 7:**
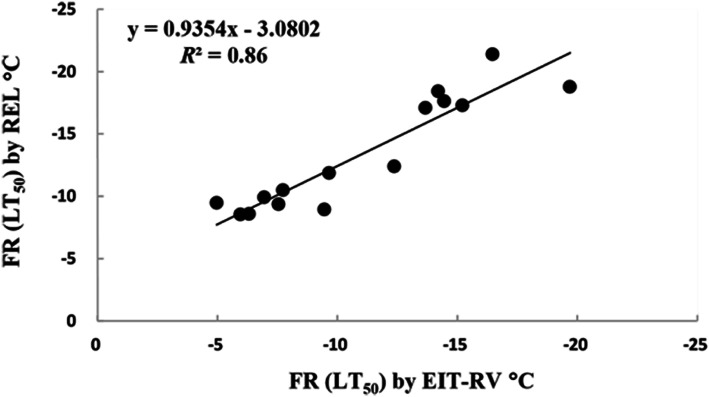
Correlation of freezing resistance (FR, LT_50_) (LT_50_, Temperature at which 50% of the plants or organs are killed) in stems of floribunda roses measured by electrical impedance tomography reconstructed values (EIT-RV) with relative electrolyte leakage (REL) method values. The significance is at *P* < 0.01. Linear correlation coefficient *r* is 0.93. The data of stems were pooled data of three floribunda rose varieties (*n* = 15). *R*^2^ is coefficient of determination.

During frost dehardening, the freezing resistance assessed by the EIT reconstructed values correlated significantly and linearly with the traditional EL method (*r* = 0.93, *P* < 0.01; Fig. 7). According to the regression equation, the determination coefficient of the EIT reconstructed value method was as high as 0.86, showing high reliability.

## Discussion

### Optimum EIT parameters for measuring freezing resistance in stems of floribunda roses

EIT reconstructs the distribution of electrical parameters in biological tissues depending on the electromagnetic characteristics of those biological tissues. The interactions of charged ions and ionic groups, among others, result in biological tissues maintaining a certain structure and functional state, and changes in the physiological state are reflected in electrical properties that are captured because of the sensitivity of the EIT system. There are considerable differences between plants and humans or animals in terms of cell structure, tissue active state, measured volume, and sensitivity of power source excitation, among other factors. When the EIT technique is applied to animals or humans, the selected excitation current and frequency are higher than those used in plants. In terms of the physical model, because the diameter of tissue measured in humans and animals is much larger than the diameter of a rose, a test device of organic glass with different diameters was constructed. When the test device radius was small, serious noise pollution affected the reconstructed image of the rose stem. In the present experimental system, the excitation mode was the opposite excitation and the measurement mode was the adjacent measurement, the optimum EIT reconstructed image was obtained by selecting a rose stem diameter more than 4 mm, with an 8-cm inner radius of the device, an excitation current frequency of 1 kHz and an amplitude of excitation current of 250 μA. According to the EIT measuring principle, increasing the number of electrodes could increase the resolution of the system and improve the imaging quality by increasing the number of independent measuring numbers. But the measured object was usually a closed domain with definite perimeter. The increase in the number of electrodes would inevitably reduce the electrode gap, limit the measuring current to penetrate into the central area, and then affect the detection sensitivity of the central area or deep targets. For the relatively small-sized imaging objectives, too many electrodes would reduce image quality instead in the case of insufficient hardware system performance. When the radius of the physical model was 8 cm and the electrode spacing was greater than 10 mm, a clearer rose stem image was detected, and the shape and location of the stem were also determined through reconstructed images. When rose stems were measured with different diameters, reconstructed images could not be generated when the measured diameter was small; however, the EIT images of stems could be detected clearly when the diameter was larger than 4 mm. Based on medical research, only objects larger than a given size can be distinguished because of the difficulty given the number of electrodes and the measurement precision of each electrode [29, 30]. This finding explains the necessity of highly sophisticated mathematical algorithms that address the inverse problem and the associated difficulty. Inter- and intra-individual differences in electrode conductivity with associated image distortion and artefacts are further difficulties in absolute EIT. The human or animal body part of interest is rarely precisely round, and inter-individual anatomy is variable, e.g., thorax shape, affecting individual electrode spacing [31], whereas the body of a rose stem is much more precisely round. To further reduce imaging errors, the use of active surface electrodes could improve the signal-to-noise ratio [32]. Consequently, the reconstructed images changed with changes in the diameter of the samples, the excitation current frequency and the magnitude of the excitation current when other conditions remained the same. The present experiment was a further original and exploratory use of EIT in the study of plant resistance.

### Application of EIT in evaluating freezing resistance in stems of floribunda roses

In the present study, the EIT reconstructed images of floribunda rose stems were obtained under freezing tests during frost dehardening. The freezing resistance estimated according to the EIT reconstructed values was close to that measured by the conventional EL method with a significant linear correlation ((*r* = 0.90, *P* < 0.01). After the freezing test, the stems were in different states, which had an important effect on the conduction of the electric current. The changes in EIT images and reconstructed values were determined by decreasing the temperature step-by-step, which might be related to the damage caused to cell membranes due to the physiological, anatomical and structural changes. Researchers believed that cells show high impedance to low frequencies (f < 1 kHz), while low-frequency currents only existed in the extracellular space, and the impedance was mainly composed of extracellular resistance [33]. In this study, the excitation current frequency of 1 kHz was applied, and the impedance was probably composed of extracellular resistance. The cell-membrane was an important medium in material exchange and information transmission between cells and external environment. With the advent of freezing injury, the permeability of the cell- membrane was changed, which resulted in the exudation of protoplast ions into the ectoplast and the enhancement of the cell viability. Thus, there was a new steady equilibrium between the ion concentrations of the symplast and the apoplast and the dielectric properties of the cell-membrane changes. With the decrease of temperature, the freezing damaged to the stem increases while the ectoplasmic resistance decreases, and this leaded to the lower resistivity than that of the control group (4 °C) and the differences in EIT imaging results. The EIT measuring speed was about 1.25 frames per second. After inserting the rod and stabilizing the salt solution, a clear EIT image was collected, and one-dimensional EIT reconstruction value was extracted from the image. The EIT technique was sufficiently sensitive to detect the changes in the resistivity of stems caused by low temperatures (the electrical resistivity changes are reflected by the reconstructed values). The electrical resistivity distribution of plant stems is more uniform than that of animal or human organs, and the EIT reconstructed image could accurately reflect the position of the stem with high quality. The functional imaging of ornamental plants was achieved. Application of the EIT technique is increasing in medical research [22–24] and will be a type of detection method that is closely related to the biological physiological functions of plants.

Plant cells are composed of intracellular fluids, cell membranes, and a cell wall and are suspended in extracellular fluids [34]. EIT has the potential to visualize tissue physiology and pathology in terms of tomographic images of the electrical impedance distribution, provides more information about tissue physiology and pathology, and hence has greater potential in several applications [29, 35, 36]. EIT measures the resistance of a plant cross-section, without consideration of plant length and cross-sectional area. EIT measurements are carried out in plant faults, and 2D measurements are used. When the excitation current and frequency reach a certain level, the current flux can distribute in most areas of the faults and improve the measurement accuracy. Another advantage of EIT is that if the methodology allows visualization of the internal structures of tree stems, important additional information can be provided on the damage caused by freezing. Further research should be continued to achieve more advantages associated with EIT technology.

## Conclusion

In conclusion, in the present experiment, a new EIT technique used in medicine was introduced for use with ornamental plants, and a functional imaging technique of plants was realized. Freezing resistance was evaluated by fitting the reconstructed value with the logistic equation within two days of the artificial freezing temperature test. The freezing resistance calculated by EIT reconstructed values was highly correlated with that obtained using the traditional EL method, although it was slightly lower than that evaluated by the EL method. The reconstructed value of EIT could be used to evaluate the freezing resistance of floribunda rose stems, showing that the EIT technique might be applied as a rapid and effective method to evaluate the freezing resistance of trees.

## Methods

### Plant materials

Two-year-old floribunda roses (*Rosa hybrida* ‘Floribunda’) of three varieties, ‘Red Cap’, ‘Tender and Soft as Water’ and ‘Carefree Wonder’, were obtained from the Chinese Rose Base Co., Ltd. (32°98′ N, 112°53′ E) located in Nanyang City, Henan Province, was the biggest base of Chinese rose produce in China. We got the permission to collect the plant samples from the Chinese Rose Base Co., Ltd., and the plant materials were formally identified by Mr. Guoyou Zhao. The roses were planted in Specimen Park (38°50′ N, 115°26′ E) on the East Campus of Hebei Agricultural University, Baoding City, Hebei Province, on 31 March 2013. The experimental design was a complete randomized block in which 35 plants per plot were planted at a spacing of 0.25 m × 0.30 m with three replications. A total of 315 plants were planted (35 plants/plot × 3 replications × 3 varieties = 315). Before the roses were planted, the soil was ploughed and barnyard manure fertilizer was applied. After planting, in spring and autumn, the soil was watered one to two times per week, while during summer, the soil was watered according to the actual conditions by paying attention to the amount of rainfall, preventing water from accumulating in the soil. In summer, the broad-spectrum fungicide carbendazim was sprayed on the rose leaves to control black spot disease (*Diplocarpon rosae*); diseased leaves were removed, and defoliated leaves were cleared timely. In the growing season, weeding was carried out artificially everyday during periods of vigorous weed growth or two to three days after watering during the rapid growth stage of roses. These management measures were applied to maintain consistent growth of the different rose varieties.

### Freezing temperature tests

The freezing resistance measurement experiment was conducted from February to May 2016 during frost dehardening. Five sampling times occurred at intervals of 20 days, on 22 February, 14 March, 4 April, 25 April, and 16 May for the floribunda rose varieties. Nine rose plants were selected for each variety that exhibited good growth. The location of each sampling was in the middle of the rose plant; four leafy branches were collected per plant. A total of 108 branches (3 plants × 3 treatments × 3 plots × 4 directions = 108) were collected [37]. Before performing the freezing tests, the samples were carefully rinsed with tap water 3 times and then with distilled water additional 3 times to remove surface pollutants. The plastic bags with samples were exposed to seven temperatures (six freezing temperatures plus one control at 4 °C) in refrigerators (BCD-252WBCS; Haier, Qingdao, China). These freezing temperatures ranged from conditions that would likely kill the samples to those that would cause no damage and were chosen according to the freezing resistance determined by previous assessments [38]. The rate of cooling was 4 °C h^− 1^. The samples were kept at target temperatures for 4 h and were then moved to 4 °C to allow gradual thawing [39, 40]. Then, the samples were used for EIT, electrical impedance spectroscopy and relative EL measurements after 2 h of thawing.

### EIT image reconstruction and data acquisition

**Fig. 8:**
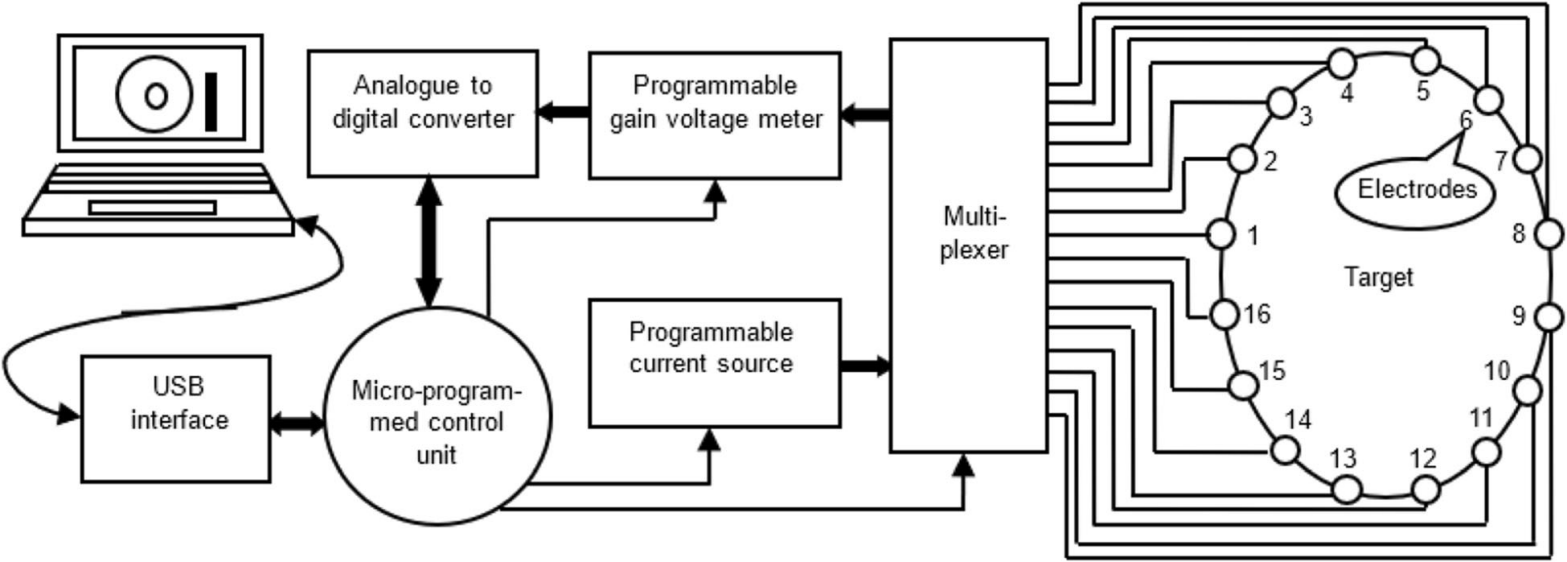
Diagram of the electrical impedance tomography (EIT) data acquisition system

For the EIT images of rose stems at different temperatures, the EIT image reconstruction was based on the data acquisition system of The Fourth Military Medical University [41], EIT Monitor image reconstruction software [42], the methods of EIT as a tool for phenotyping plant roots [14], and a real-time EIT imaging system based on the split augmented Lagrangian shrinkage algorithm [34]. The data acquisition system included a multiplexer, analogue to digital converter, and micro-programmed control unit, among other components [41] (see Fig. 8). The driving mode of the system was quasi-opposite direction drive, the excitation source was produced by digital synthesis mode, the analogue-digital converter was integrated into the system with high speed and high precision, and the USB interface between the system and the host computer was high-speed and was easily plugged in. The software system primarily completed the functions of the interface with the hardware system, the simulation imaging, the measured data imaging, the image processing, and analysis of the results, among other functions. The weighted damped least square algorithm was used to reconstruct EIT images [43], and 16 electrodes were used for continuous imaging. The imaging speed was approximately 1.25 frames per second.

A concentration of 0.9% physiological saline was used as the background (impedance did not change with the frequency) [43]; the rose stem was placed 20 mm from the edge of the plexiglass container, and a stem length of 70–90 mm was inserted vertically. After the liquid surface was completely calm, approximately 10 data frames were collected for imaging. The boundary voltage was measured with current as excitation. The image was weighted by the least square method through the boundary voltage at different frequencies. Then the reconstructed value of boundary voltage was obtained. The results of EIT imaging were displayed on a relative grey scale. When the reconstructed value of the obtained image was less than 0, the resistivity of the corresponding region was reduced, and in the pseudo-colour image, the side-coloured red was represented, and the pure red part of the image corresponded to the minimum value of the EIT reconstruction result. When the reconstructed value was greater than 0, the resistivity of the corresponding region was increased, with the blue-coloured side represented, and the pure blue part corresponded to the maximum value of the reconstruction result. The median value of the reconstruction result corresponded to the pure green colour in the image. Larger absolute reconstructed values in the same location indicated greater variation in the resistivity in that region [37].

### Relative EL measurement

Electrolyte osmosis method (EL) was used to determine membrane permeability. After the freezing test, four stems, each 10 mm in length, were cut from the middle of the stems. Twelve millilitres of distilled water were added to each test tube, which was then shaken at room temperature (22 °C) for 24 h before the first conductivity measurement (C_1_) and the blank measurement (C_*blank* 1_) (only distilled water was measured). The samples were then heat-killed at 100 °C for 20 min and shaken for another 24 h before the second conductivity measurement (C_2_) and the blank measurement (C_*blank* 2_). The relative electrolyte leakage (REL) was defined as [44]:

1 $$ REL=\frac{C_1-{C}_{blank1}}{C_2-{C}_{blank2}}\times 100\% $$

For the EL method, the total number of stem samples used was 1680 (4 stem samples × 4 replicates × 7 temperatures × 3 varieties × 5 times = 1680).

### Freezing resistance calculation by means of EIT reconstructed values and EL method values

To determine freezing resistance, the EIT reconstructed values and REL values were modelled using a logistic sigmoid function (in Eq. 2) [44, 45] with respect to the tested temperatures:

2 $$ y=\frac{A}{1+{\mathrm{e}}^{B\left(C-x\right)}}+D $$

where *x* is the tested temperature (°C), *y* is the EIT reconstructed value (or REL value), *B* is the slope at the inflection point *C* (Ωm °C^− 1^), *C* is the inflection point of the function representing the freezing resistance value (i.e. semi-lethal temperature LT_50_, °C), and *A* and *D* define asymptotes of the function [33, 38]. *A* + *D* denotes the EIT reconstructed value with no freezing injury (or the maximum value of REL under freezing injury); *D* represents the minimum EIT reconstructed value under freezing injury (or the basic value of REL with no freezing injury) [46–48].

### Statistical analyses

The calculation method of the EIT image reconstructed values was described in the section of 5.3. The relationship of the freezing resistance in stems evaluated by the EIT and EL methods after exposure to freezing tests was studied by linear regression analysis. The original data (the means of each variety at each given time) from all three varieties over the entire study period were pooled, and linear regression curve fit was applied (*SPSS v22.0* statistical software package for Windows, *SPSS* Inc., Chicago, IL, USA). For the evaluation of the reliability and accuracy of the models, the coefficient of determination (*R*^2^) was examined. The correlation coefficient of linear regression (*r*) was also provided. Repeated measurement analysis was applied for analysing the differences in freezing resistance of stems among different methods. The differences among different assessment methods for the LT_50_ estimates for each time instant were considered significant when the values of the Wald 95% confidence intervals did not overlap. The correlation of freezing resistance estimated by EIT reconstructed values with values of the EL method used pooled data of the three varieties.

## Data Availability

All data sustaining the results in this study are included in this article or its information files. Other datasets generated during this study are available upon reasonable request from the corresponding author (Ji Qian).

## Abbreviations

EIT: Electrical Impedance Tomography
2D: Two-Dimensional;
EL: Electrolyte Leakage
LT50: Semi-lethal Temperature
